# Supplementary figures and images for: Human Schistosoma exposure risk in rice fields and an exploration of fish species for snail and schistosomiasis biocontrol
Source: PLOS Glob Public Health. 2025 Jun 11;5(6):e0004726. doi: 10.1371/journal.pgph.0004726 (PMC12157053; doi:10.1371/journal.pgph.0004726)

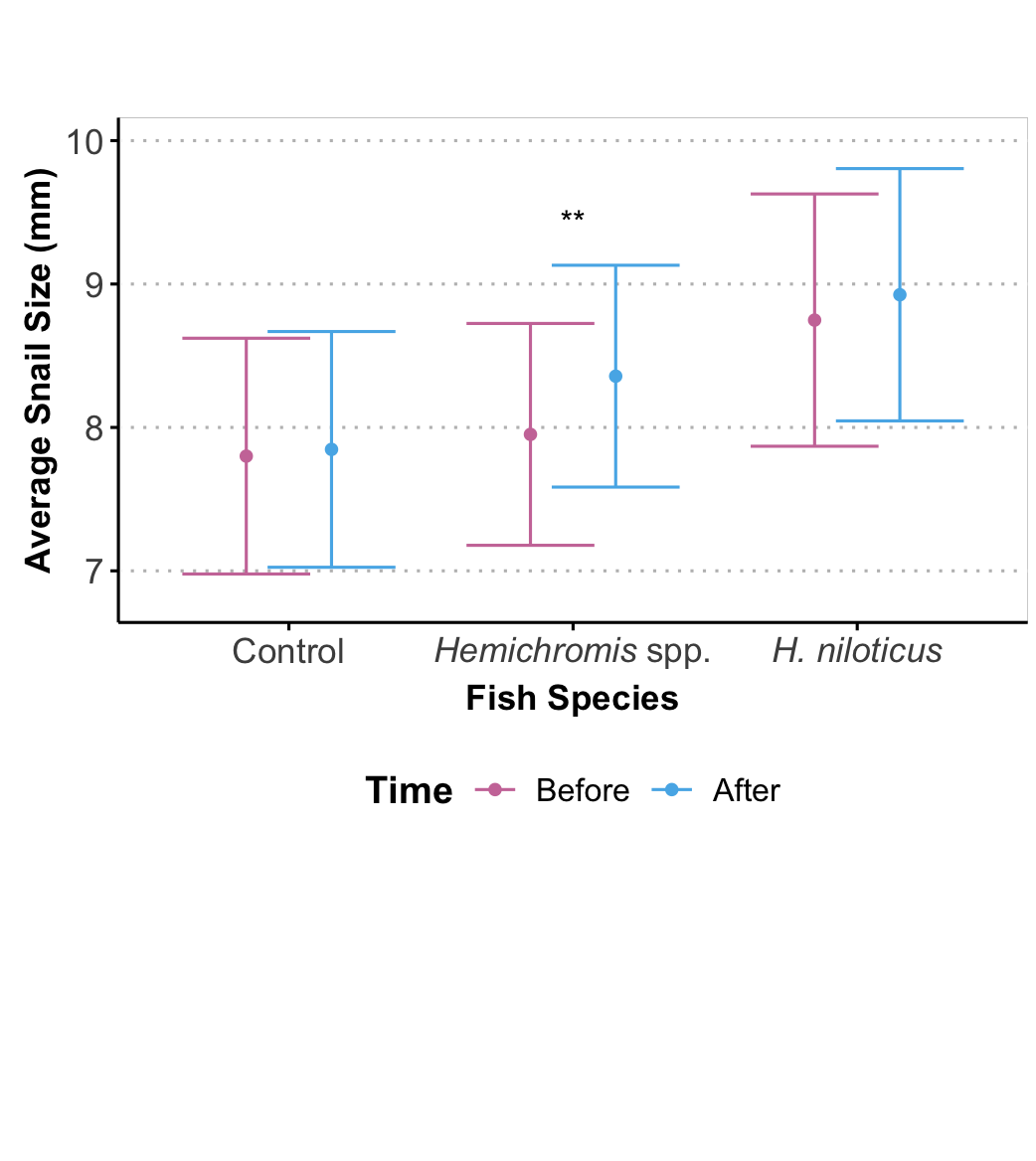

Supplement: S1 Fig — Only snails exposed to the smallest of the predators, Hemichromis spp., were larger after predation than before. ** = p-value ≤0.05 and ≥0.01. (TIFF) [file pgph.0004726.s001.tiff]
