# Supplementary material for: Human Schistosoma exposure risk in rice fields and an exploration of fish species for snail and schistosomiasis biocontrol
Source: PLOS Glob Public Health. 2025 Jun 11;5(6):e0004726. doi: 10.1371/journal.pgph.0004726 (PMC12157053; doi:10.1371/journal.pgph.0004726)
Supplement: S1 Table — Tank and temporal block were included as random intercepts. (DOCX) [file pgph.0004726.s002.docx]

**Supplemental Table 1.** Avoidance Behavior (Above and Below Water Line) contrasts between snail species across Fish Species chemical cues.

| **Snail spp. Contrast** | **Fish Species Chemical Cue** | **Vegetation** | **Odds Ratio** | **SE** | ***p* value** |
| --- | --- | --- | --- | --- | --- |
| *Bi. pfeifferi* – *B. truncatus/globosus* | Control | Absent | 2.54 | 0.87 | 0.018 |
| *Bi. pfeifferi* – *Lymnaea* spp. | Control | Absent | 1.64 | 0.52 | 0.263 |
| *B. truncatus/globosus* – *Lymnaea* spp. | Control | Absent | 0.65 | 0.23 | 0.438 |
| *Bi. pfeifferi* – *B. truncatus/globosus* | Control | Present | 1.36 | 0.38 | 0.514 |
| *Bi. pfeifferi* – *Lymnaea* spp. | Control | Present | 0.87 | 0.23 | 0.862 |
| *B. truncatus/globosus* – *Lymnaea* spp. | Control | Present | 0.64 | 0.18 | 0.241 |
| *Bi. pfeifferi* – *B. truncatus/globosus* | *C. gariepinus* | Absent | 0.90 | 0.29 | 0.945 |
| *Bi. pfeifferi* – *Lymnaea* spp. | *C. gariepinus* | Absent | 0.49 | 0.15 | 0.041 |
| *B. truncatus/globosus* – *Lymnaea* spp. | *C. gariepinus* | Absent | 0.54 | 0.16 | 0.087 |
| *Bi. pfeifferi* – *B. truncatus/globosus* | *C. gariepinus* | Present | 1.00 | 0.65 | 1.00 |
| *Bi. pfeifferi* – *Lymnaea* spp. | *C. gariepinus* | Present | 0.30 | 0.16 | 0.063 |
| *B. truncatus/globosus* – *Lymnaea* spp. | *C. gariepinus* | Present | 0.30 | 0.16 | 0.063 |
| *Bi. pfeifferi* – *B. truncatus/globosus* | *Hemichromis* spp. | Absent | 1.10 | 0.28 | 0.925 |
| *Bi. pfeifferi* – *Lymnaea* spp. | *Hemichromis* spp. | Absent | 1.54 | 0.40 | 0.216 |
| *B. truncatus/globosus* – *Lymnaea* spp. | *Hemichromis* spp. | Absent | 1.41 | 0.37 | 0.395 |
| *Bi. pfeifferi* – *B. truncatus/globosus* | *Hemichromis* spp. | Present | 1.00 | 1.42 | 1.00 |
| *Bi. pfeifferi* – *Lymnaea* spp. | *Hemichromis* spp. | Present | 0.092 | 0.097 | 0.062 |
| *B. truncatus/globosus* – *Lymnaea* spp. | *Hemichromis* spp. | Present | 0.092 | 0.097 | 0.062 |
| *Bi. pfeifferi* – *B. truncatus/globosus* | *H. niloticus* | Absent | 0.49 | 0.17 | 0.084 |
| *Bi. pfeifferi* – *Lymnaea* spp. | *H. niloticus* | Absent | 0.53 | 0.18 | 0.16 |
| *B. truncatus/globosus* – *Lymnaea* spp. | *H. niloticus* | Absent | 1.10 | 0.34 | 0.95 |
| *Bi. pfeifferi* – *B. truncatus/globosus* | *H. niloticus* | Present | 1.55 | 0.85 | 0.701 |
| *Bi. pfeifferi* – *Lymnaea* spp. | *H. niloticus* | Present | 1.32 | 0.69 | 0.861 |
| *B. truncatus/globosus* – *Lymnaea* spp. | *H. niloticus* | Present | 0.85 | 0.49 | 0.955 |
| *Bi. pfeifferi* – *B. truncatus/globosus* | *O. niloticus* | Absent | 1.15 | 0.28 | 0.823 |
| *Bi. pfeifferi* – *Lymnaea* spp. | *O. niloticus* | Absent | 1.83 | 0.45 | 0.040 |
| *B. truncatus/globosus* – *Lymnaea* spp. | *O. niloticus* | Absent | 1.58 | 0.40 | 0.154 |
| *Bi. pfeifferi* – *B. truncatus/globosus* | *O. niloticus* | Present | 0.48 | 0.30 | 0.475 |
| *Bi. pfeifferi* – *Lymnaea* spp. | *O. niloticus* | Present | 0.15 | 0.086 | 0.0024 |
| *B. truncatus/globosus* – *Lymnaea* spp. | *O. niloticus* | Present | 0.32 | 0.14 | 0.023 |
