# Supplementary material for: Human Schistosoma exposure risk in rice fields and an exploration of fish species for snail and schistosomiasis biocontrol
Source: PLOS Glob Public Health. 2025 Jun 11;5(6):e0004726. doi: 10.1371/journal.pgph.0004726 (PMC12157053; doi:10.1371/journal.pgph.0004726)
Supplement: Attachment — Submitted filename: Response to Reviewers-AS&ES-April27.docx [file pgph.0004726.s004.docx]

Dear Reviewers,

Thank you both for your comments. We have responded to specific questions and comments below. However, there were comments made by Reviewer 2 that were not detailed enough for us to be certain that we are adequately addressing their concerns. We did our best to attempt to address those comments but are happy to make additional revisions if this reviewer is willing to further clarify their comments. Thank you for your comments and time.

Under Reviewer's Responses to Questions

1. We have addressed the data sharing concern and have added a copy of the data used for the field and two experiments on Figshare at <https://figshare.com/articles/dataset/Data_for_Field_Surveys_and_Experiments-Sack_Selland_xlsm/28861280?file=53963564>.

Under Review Comments to the Author

2. Reviewer #1: The authors are to be commended for an interesting, thoughtful and scholarly manuscript that brings data on schistosome and molluscan host distribution together with novel information on interactions between snails and potential fish predators. I have no major concerns with the methodology, analysis of the data, and conclusions reached, although the manuscript would be improved if some minor issues are addressed:
   a. I am curious whether there is any data on the seasonality of human schistosome infection in the area, and whether this correlates with rice farming activities.
      i. In the region, infection in children generally increases over the rainy season as treatment of praziquantel is traditionally administered during the dry season (likely due to access issues). However, there is no information specific to rice farming families and this is part of the ongoing work for one of the first authors. The following sentence has been added to indicate ongoing research on this point: “Additionally, ongoing work seeks to construct direct linkages between rice fields and *Schistosoma* infection in rice farming communities, which will shed further light on the exposure risk to farmers.”
   b. As snail-fish trophic interactions are a major theme of the manuscript, it would benefit from at least some background information on how snails detect predatory fish and what avoidance behaviors are employed to avoid predation. Is there anything known of the molecular nature of fish signals and how snails detect these signals?
      i. We added the content at the end of the paragraph below to address the reviewer’s suggestion.
      ii. “If rice farming represents an occupational risk of schistosomiasis, snail control using molluscicides would be problematic in rice fields because molluscicides are often toxic to humans and wildlife [4, 7], and thus alternative approaches to snail control would likely be necessary in rice fields. One viable approach suggested by researchers and the WHO is the introduction of snail predators, such as fish or crayfish [8]. Fish have been used to control non-Schistosoma-transmitting snail species that are pests in rice fields in Asia as part of a practice called rice-fish co-culturing. This practice, however, has not been tested as a control for Schistosoma-transmitting snails. However, the efficacy of any biocontrol mechanism is often complex, depending on the foraging and competitive abilities of the biocontrol agent and the anti-predator or anti-competitor responses of the snails, among other things [9, 10]. For example, snails can detect predator chemical cues (kairomones) [11] and respond with antipredator behaviors, such as predator avoidance and refuge use, and some snails can even display predator-species-specific responses [12, 13]. Predator avoidance often entails moving away from high risk locations and moving towards or above the water line, whereas refuge use often involves moving into dense vegetation.”
   c. Line 126 – how confident are the authors in their identification of cercariae by microscopy alone? Were any molecular analyses employed to confirm species identification? Numerous Schistosoma species are found in West Africa – several species of veterinary importance in addition to the human pathogens are found in Senegal and neighboring countries, and hybridization between some species has been well documented.
      i. This is an important point, thank you. We do not use molecular analyses to confirm molecular cercariae identification and thus do risk false positives with other *Schistosome* species. This region of Senegal specifically does have well documented occurrence of *Schistosoma* hybrids, as you mentioned. We have added this limitation to our discussion section. We added the following to the Discussion to address this concern.
      ii. “However, we acknowledge that only morphological identification of Schistosoma cercariae species was conducted and thus it is possible that some of the Schistosoma were not cercariae that infect humans. Nevertheless, researchers using morphological identification are highly trained, and there is documented evidence that individuals working in rice fields urinate and defecate in and around rice fields [50], which would expose susceptible snails to human-Schistosoma species. Additionally, ongoing work seeks to construct direct linkages between rice fields and Schistosoma infection in rice farming communities, which will shed further light on the exposure risk to farmers.”
   d. Line 123 – “Bulinus foskalii” should be “Bulinus forskalii”. Also see labeling of Figure 1.
      i. This has been fixed, thank you
   e. Line 212 – define “AICc” (Akaike information criterion)
      i. This has been fixed, thank you
   f. Labeling in the figures is small and hard to read, even when viewing the downloaded hi-res TIFF files. Suggest redoing all the labeling in a larger font.
      i. All fonts have been fixed to make them easier to read, thank you
2. Reviewer #2: • The rationale and knowledge gap for the study should be more clearly articulated.
   i. The Introduction has been re-written/organized to better establish the rationale and knowledge gap.
   b. A section outlining the overall study design is currently missing and should be included.
      i. This was covered from 351-363 in the introduction, and we think this provides a clear outline for the reader to understand the study design.
   c. A data analysis section summarizing the statistical methods used is needed. The models described for each experimental procedures could be included in this section.
      i. This was covered under each of the three sections and not as a single statistical methods section. We think this is easier for the reader to follow instead of putting all the statistical methods together. For the field methods, this is line 457-465, experiment 1 – line 509-519 and experiment 2, 538-543.
   d. The description of the experimental approach could be more concise by referencing prior studies that detail predator avoidance and selective predation procedures.
      i. We appreciate the reviewer’s comment, but the fish husbandry specifics were requested to be included specifically by the first editor to understand animal welfare. Furthermore, as we did this work in Senegal, the housing and methods were not identical to those used by work in laboratory conditions in the US.
   e. The discussion section lacks focus and depth in some areas, which weakens the overall argument of the paper.
      i. We have attempted to provide greater focus and depth to the Discussion, but without more specific details, it is challenging to know if we have adequately addressed the reviewer’s concern. Both the Introduction and the Discussion were revised to improve clarity, focus and depth.
   f. Including a paragraph that summarizes the strengths and limitations of the study would be valuable.
      i. Thank you for the comment, this has been added to the end of the discussion. The paragraph is also below.
      ii. “Overall, this work confirms an occupational risk of schistosomiasis to rice famers because snails shedding Schistosoma cercariae were regularly found in rice fields and neighboring irrigation canals across seasons and years, suggesting that snail control in rice fields might be worthwhile. However, we acknowledge that only morphological identification of Schistosoma cercariae species was conducted and thus it is possible that some of the Schistosoma were not cercariae that infect humans. Nevertheless, researchers using morphological identification are highly trained, and there is documented evidence that individuals working in rice fields urinate and defecate in and around rice fields [50], which would expose susceptible snails to human-Schistosoma species. Additionally, ongoing work seeks to construct direct linkages between rice fields and Schistosoma infection in rice farming communities, which will shed further light on the exposure risk to farmers. For the avoidance and predation experiment, we attempted to best replicate natural conditions by using wild caught, native snails and fish and plants directly collected from water sources. However, for the avoidance experiment, we only tested hourly, and some immediate, short-term avoidance behaviour may have been missed. For the predation experiment, we only provided snails as food and thus preferences for snails versus other food items remains equivocal. Ongoing work in actual rice fields will facilitate capturing these more complex and natural systems.”
   g. Significant formatting revisions are required to meet publication standards, particularly regarding font size, spacing, and table presentation.
      i. Thank you, the tables have been reformatted and are inserted in the manuscript after first reference, as the journal requests. There is not a specific font size requested, and all are size 11 and the one with a return before the parenthesis has now been removed.
   h. Substantial revisions to the manuscript’s language are needed to improve clarity, coherence, and overall flow.
i. We have attempted to improve clarity, coherence, and flow in both the Introduction and Discussion. The Introduction, in particular, was very heavily revised. However, without more specific details, it is challenging to know if we have adequately addressed the reviewer’s concern.
